# Bibliometric Review on New Possibilities of Antimycobacterial Agents: Exploring Siderophore Desferrioxamine’s Applications as an Antimicrobial Agent

**DOI:** 10.3390/ph16091335

**Published:** 2023-09-21

**Authors:** Patrícia Vieira de Oliveira, Roseane Lustosa de Santana Lira, Rafael de Abreu Lima, Yasmim Costa Mendes, Antenor Bezerra Martins, Bruna de Oliveira de Melo, Millena Ferreira Goiano, Rivaldo Lira Filho, Flávia Baluz Bezerra de Farias Nunes, Amanda Silva dos Santos Aliança, Wellyson da Cunha Araújo Firmo, Rafael Cardoso Carvalho, Adrielle Zagminan, Eduardo Martins de Sousa

**Affiliations:** 1 Graduate Program in Microbial Biology, CEUMA University—UNICEUMA, São Luís 65075-120, Brazil; patricia001444@ceuma.com.br (P.V.d.O.); yasmim001407@ceuma.com.br (Y.C.M.); amanda.alianca@ceuma.br (A.S.d.S.A.); wellyson004830@ceuma.com.br (W.d.C.A.F.); adrielle004602@ceuma.com.br (A.Z.); 2 Graduate Program in Health Sciences, Federal University of Maranhão—UFMA, São Luís 65080-805, Brazil; roseane.lustosa@discente.ufma.br (R.L.d.S.L.); rafael.al@ufma.br (R.d.A.L.); carvalho.rafael@ufma.br (R.C.C.); 3 Graduate Program in Health and Services Management, CEUMA University—UNICEUMA, São Luís 65075-120, Brazil; antenor_oficial@hotmail.com; 4 Graduate Program in Biodiversity and Biotechnology—BIONORTE Amazonian Network, Federal University of Maranhão—UFMA, São Luís 65080-805, Brazil; brunaoliv.96@gmail.com; 5 Graduate Program in Biomedicine, CEUMA University—UNICEUMA, São Luís 65075-120, Brazil; mgoiano130@gmail.com; 6 Graduate Program in Nursing, St. Therese College—CEST, São Luís 65045-180, Brazil; rivaldolirafilho@gmail.com; 7 Graduate Program in Nursing, Federal University of Maranhão—UFMA, São Luís 65080-805, Brazil; flavia.farias@ufma.br

**Keywords:** siderophore, desferrioxamine, anti-infective agents, *Mycobacterium*

## Abstract

Mycobacteria cause tuberculosis and other serious diseases. Understanding their mechanisms of resistance to our immune system and exploring novel drugs are critical strategies to combat infections. A bibliometric analysis was performed to identify publication trends and critical research areas in the field of the antimicrobial activity of desferrioxamine. A total of twenty-four publications on the topic, from 2012 to 2023, were retrieved from databases including Web of Science, Scopus, PubMed, and Embase, using specific keywords. The quality of the publications was assessed using impact and productivity metrics, with an average annual publication rate of 2.1 articles. The United States emerged as the most productive country, with medicine (23.4%, 11 publications) and biochemistry, genetics, and molecular biology (21.3%, 10 publications) as the top research fields. The five most cited publications accounted for 672 citations, with a relatively low h-index (11:11). In conclusion, there has been a lack of publications on this topic in the last decade. The United States dominates production and publication in this area, and there appears to be limited exchange of knowledge, ideas, and technology within the field. Therefore, fostering international cooperation through funding is essential to facilitate further research and development of desferrioxamine-related studies.

## 1. Introduction

Bacteria of the genus *Mycobacterium* are responsible for various diseases affecting the respiratory system and other organs in humans and animals and include more than 200 species [1]. These microorganisms can be found mainly in soil and water, but most do not cause infection or disease and are considered apathogenic [2]. Among the pathogenic bacteria, we have two groups. One is the *Mycobacterium tuberculosis* complex (MTBC), formed by *M. tuberculosis* (Mtb) along with seven other similar mycobacteria: *M. bovis*, *M. africanum*, *M. microti*, *M. caprae*, *M. pinnipedii*, *M. canetti*, and *M. mungie* [3], which are pathogens capable of causing tuberculosis (TB), one of the main causes of death from infectious diseases in the world, surpassed only by COVID-19 [4].

Other mycobacterial infections are caused by non-tuberculous mycobacteria (NTM), which include species of the *Mycobacterium abscessus* complex (MABSC), characterized as rapidly growing mycobacteria [3]. This complex includes related species: *M. abscessus*, *M. massiliense*, and *M. bolletii* [5]. These bacteria can cause chronic pulmonary disease, post-traumatic wound infection, and disseminated cutaneous disease, mainly in immunocompromised patients, and are very common among patients with cystic fibrosis, according to clinical data [2].

The currently recommended treatment for TB patients is a six-month regimen of four first-line drugs: isoniazid, rifampicin, ethambutol, and pyrazinamide. The success rate of treatment is at least 85% [6]. The species possess intrinsic and acquired resistance mechanisms, making them some of the most problematic microorganisms to treat, with therapy often extending over months or even years, with the risk of antibiotic toxicity and a high rate of treatment failure [2]. The antimicrobial resistance of these mycobacteria is related to their composition and lipophilic structure, as mycobacteria have a cell wall that confers intrinsic resistance to several antibiotics. This lipophilic property creates a physical barrier that prevents proper diffusion of antibacterial drugs through the cell wall, thereby reducing the efficacy of antibiotics against these mycobacteria [6].

Drug-resistant TB represents a serious threat to global public health, hindering TB case control worldwide. Multidrug-resistant tuberculosis (MDR-TB) is defined as a clinical form resistant to rifampicin and isoniazid, while extensively resistant tuberculosis (XDR-TB), in addition to resistance to rifampicin and isoniazid, also exhibits resistance to fluoroquinolone and a second-line injectable drug such as amikacin, capreomycin, or kanamycin [7].

The search for compounds with novel mechanisms of action and physicochemical properties that enhance intracellular accumulation or act synergistically with other antimycobacterial drugs may represent a strategy to reduce and prevent further drug resistance [8]. Consequently, recent research has explored non-antibiotic antimicrobial agents as an alternative to current drugs [9]. In this context, metallophores and siderophores can be reported. Metallophores are secondary metabolites secreted by bacteria that play an important role in their virulence [10]. They are low-molecular-weight molecules produced under the conditions of scarce availability of essential transition metal ions such as iron. To support this iron deficiency, bacteria have developed various mechanisms for obtaining iron from the host since it is important for their colonization during infection. These mechanisms include the acquisition of heme-bound iron, absorption by membrane-bound uptake systems, and secretion of siderophores [11].

Siderophores, high-affinity iron metallophores, are essential pathogenicity factors in bacteria. There are four distinct types of siderophores: catecholate, phenolate, hydroxamate, and carboxylate, classified according to their iron-chelating portions. Siderophores are a type of chelating agent that can sequester iron and prevent its absorption by pathogenic microorganisms [12].

One alternative is host-directed therapies (HDTs), such as the use of the iron chelator subtype desferrioxamine (DFO) B, as studies have shown that it can be used as an adjuvant therapy with the combination of antimicrobials [13,14]. DFO is a drug that binds iron and aluminum and is produced by removing the trivalent iron portion of ferrioxamine B, an iron-containing sideramine produced by the actinomycete *Streptomyces pilosus*. This chelating agent is known for its high binding affinity for trivalent iron and lower affinity for other metals [15]. HDTs, combined with antimicrobial agents, may favor more effective elimination of bacteria, thus reducing treatment time, which may improve adherence, preventing the emergence of these rapidly growing drug-resistant mycobacteria and reducing the adverse effects caused by prolonged use of antimicrobials [14].

Given the above, we propose a bibliometric study, as such investigations are fundamental for assessing the flow of science and information and determining the state of the art or knowledge [16]. Bibliometric indicators have become essential as they provide crucial information on the number of authors, studies, countries, scientific connections, journals, and impact factors of existing publications within each product category. We acknowledge that studies correlating the antimicrobial activity of desferrioxamine remain scarce, with many gaps to be filled. Therefore, this bibliometric review is necessary to assess the quantity and quality of indexed publications on the antimicrobial and biological activities of desferrioxamine.

## 2. Results

Forty-one studies were obtained from searches in the Scopus, Web of Science (WOS), CINAHL, EMBASE, WHO, and Pubmed/Medline databases. After the studies were refined based on a temporal filter (2012 to June of 2023), document type (articles), and language (English), a total of twenty-four studies remained. All articles included in the research were then subjected to bibliometric data analysis.

### 2.1. Articles on Desferrioxamine and Predictors of Siderophores and Antimicrobial Agents: Year, Authors, Title, and Journal

The main data from the 24 publications are presented in Table 1. As shown, the yearly publication count was limited. Nevertheless, the number of publications experienced growth in 2022 (N = 6, representing 25% of the total), indicating an increasing interest among researchers in investing more in this field in recent years.

### 2.2. Analysis of the Annual Number of Publications, Main Journals, and Regions/Countries

**Figure 1:**
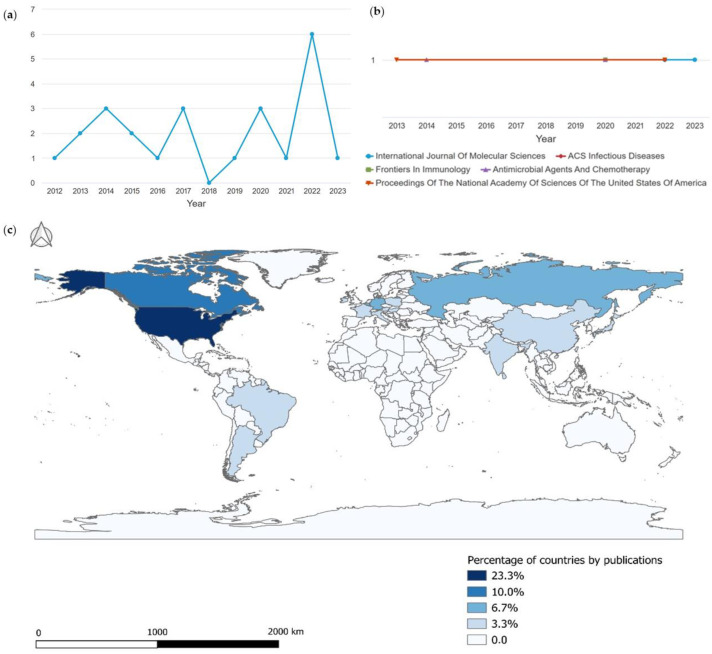
(**a**) Number of annual publications. (**b**) Most productive journals. (**c**) Countries/regions with the highest publication output on the antimicrobial activity of desferrioxamine from 2012 to June of 2023.

Figure 1a displays the number of publications on the antimicrobial activity of desferrioxamine over the years, revealing a low count in most years, with an absence in 2018 and a peak in 2022. The average number of articles published in journals during the studied period is 2.1 publications per year. The publications retrieved in this study were distributed among 21 journals, and the journals displaying greater interest in the subject are depicted in Figure 1b.

Regarding the regions/countries of the 24 publications included in the review, the majority originated from the United States, accounting for 23.4% (seven publications), followed by Canada with 10% (three publications). Belgium, Germany, Russia, and the United Kingdom contributed 6.7% each (two publications each), while countries such as Argentina, Austria, Brazil, and China each presented one publication, representing 3.3% each, as demonstrated in Figure 1c.

### 2.3. Analysis of the Main Funding and Affiliated Institutions

**Figure 2:**
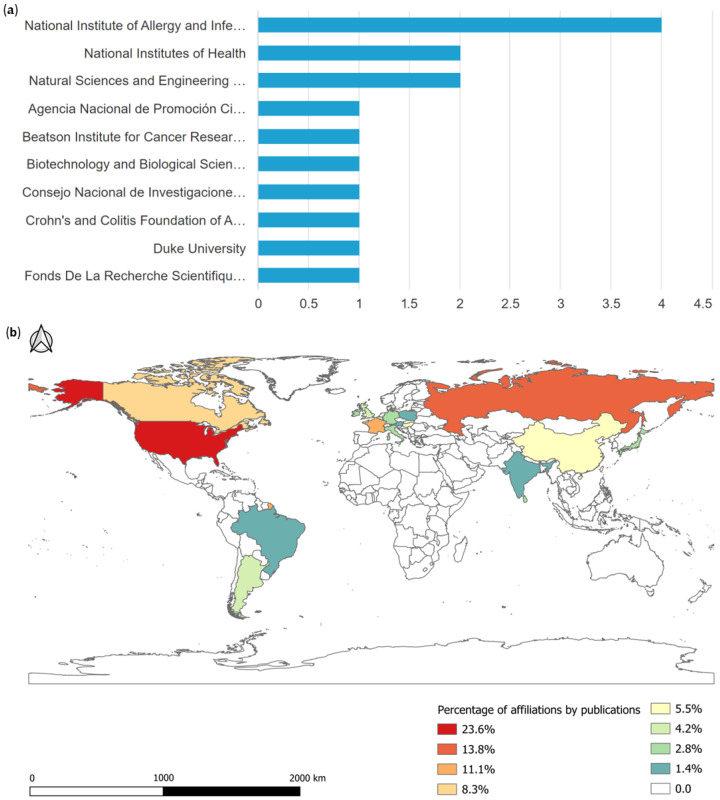
Publications by funding institutions (**a**) and affiliated institutions (**b**) conducting research on the antimicrobial activity of desferrioxamine from 2012 to June of 2023.

A total of 34 funding agencies were active in the area, and the top 10 funding agencies for research in this area were identified. The National Institute of Allergy and Infectious Diseases funded the largest number of research projects, as shown in Figure 2a. A total of 72 institutions contributed to the topic based on their region/country of origin, as shown in Figure 2b. Most of these affiliations (23.6%) were from the United States, followed by the Russian Federation (13.8%), France (11.1%), and China and Hungary (5.5% each, with four affiliations each). The United Kingdom, Belgium, and Argentina each had 4.2% (three members each), while Italy, Sri Lanka, Ireland, Germany, and Japan each had 2.8% (two members each). Finally, Brazil, Poland, and Austria had only one affiliation each, representing 1.4%.

### 2.4. Analysis of Document Types and the Most Prolific Areas of Publication

**Figure 3:**
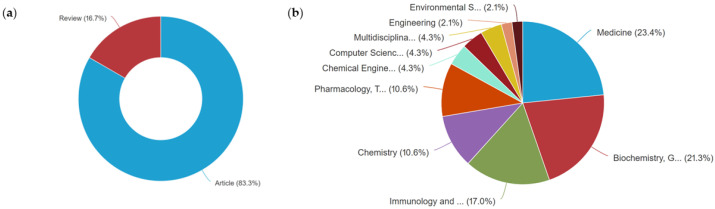
Publications by document type (**a**) and publication by area (**b**) on the antimicrobial activity of desferrioxamine, from 2012 to June of 2023.

Regarding document types, Figure 3a reveals the identification of 4 reviews and 20 original articles. Notably, the prominent areas of publication include medicine (23.4%, 11 publications), biochemistry, genetics, and molecular biology (21.3%, 10 publications), immunology and microbiology (17.0%, 8 publications), chemistry and pharmacy, pharmacology, and toxicology (10.6%, 5 publications each), as shown in Figure 3b.

### 2.5. Analysis of Citations and Index Analysis

Table 2 displays the number of citations per year for each publication, and the cumulative citations of the top 10 publications from 2012 to 2023 amounted to 672 citations. Additionally, we highlight the five most cited journals along with their respective indicators: *MBio* (244 citations, CiteScore 10.8/SjR 2.283/SNIP 1.357), *Trends in Pharmacological Sciences* (114 citations, CiteScore 23.3/SjR 2.964/SNIP 2.373), *Journal of Leukocyte Biology* (55 citations, CiteScore 11.0/SjR 1.622/SNIP 1.219), *Proceedings of the National Academy of Sciences of the USA* (45 citations, CiteScore 19.2/SjR 4.026/SNIP 2.765), and *Antimicrobial Agents and Chemotherapy* (43 citations, CiteScore 10/SjR 1.415/SNIP 1.175).

In the analysis of the top 10 institutions, it is evident that the majority are located in developed countries such as the USA, the United Kingdom, Canada, and Belgium. Notably, the institution with the highest number of publications, citations, and the best h-index is the National Institute of Allergy and Infectious Diseases (4 journals/345 citations/h-index 3). On the other hand, the National Institutes of Health had two published journals with only 19 citations, and the Natural Sciences and Engineering Research Council of Canada with two publications, had merely nine citations. In contrast, the institutions Biotechnology and Biological Sciences Research Council and Beatson Institute for Cancer Research, both from the United Kingdom, had only one publication each; however, they garnered more than 20 citations each (Table 3).

### 2.6. Analysis of Indicators in Terms of Citation and Impact Factor

**Figure 4:**
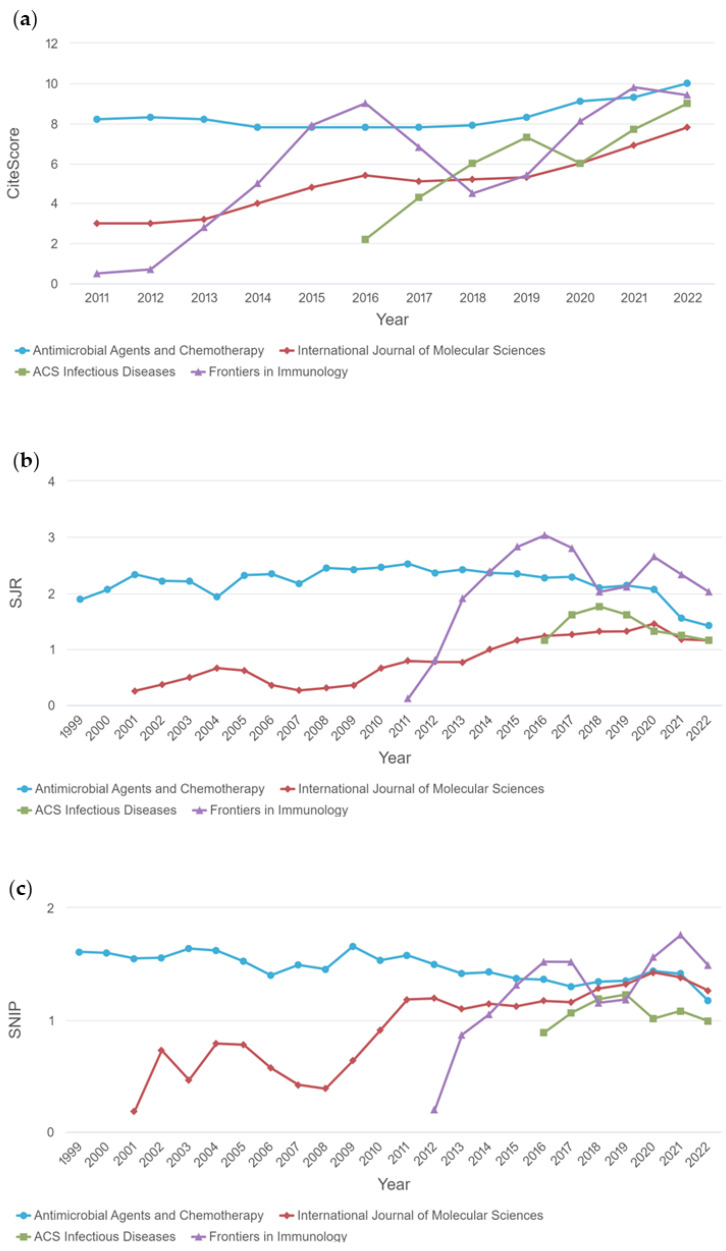
Indicators of publications on the antimicrobial activity of desferrioxamine, from 2012 to June of 2023. (**a**) Illustrates of CiteScore. (**b**) Illustrates of SJR, and (**c**) Illustrates of SNIP. The graphs compared in terms of citations and impact factor related to siderophore–desferrioxamine–antimicrobial research.

Figure 4 illustrates the CiteScore (a), SjR (b), and SNIP (c) graphs compared in terms of citations and impact factor related to siderophore–desferrioxamine–antimicrobial research, demonstrating that the journals *Proceedings of the National Academy of Sciences of the USA*, *Frontiers in Immunology*, and *Antimicrobial Agents and Chemotherapy* achieved the highest scores and metrics over the years.

The CiteScore is a straightforward measure of citation impact for sources such as journals. It is based on the number of citations received by documents in a journal over four years, divided by the number of the same types of documents indexed in Scopus and published in those same four years. The SCImago Journal Rank (SjR) graph expresses the average number of weighted citations received in the selected year by documents published in the chosen journal in the preceding three years. On the other hand, the Source Normalized Impact per Paper (SNIP) graph provides a corrective metric to account for differences in citation potential across different fields.

**Figure 5:**
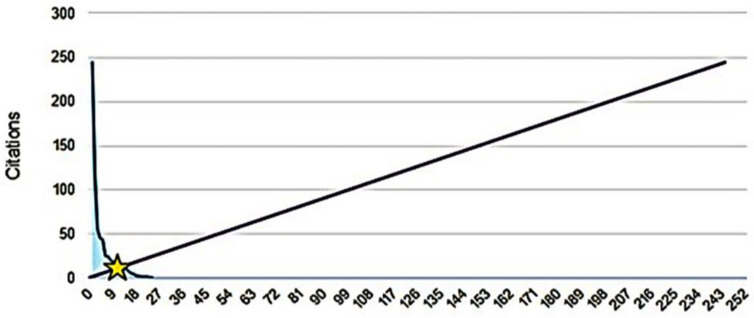
h-index of publications on the antimicrobial activity of desferrioxamine, from 2012 to June of 2023.

**Figure 6:**
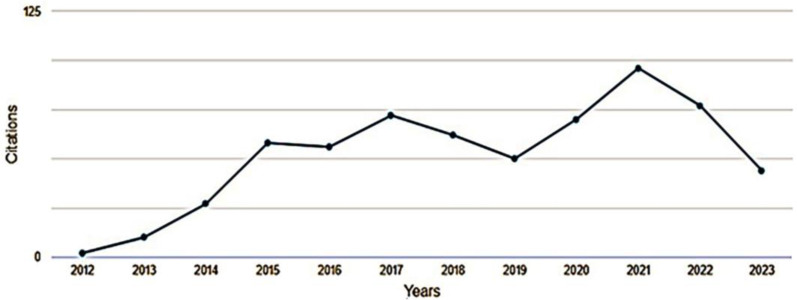
Citations of publications, over an 11-year period, on the antimicrobial activity of desferrioxamine, from 2012 to June of 2023.

The graph below depicts a 45-degree line (Figure 5), representing a 1:1 relationship between publications and citations, highlighting that out of the 24 documents considered for the h-index, 11 were cited at least 11 times (Figure 5). A comprehensive view of the selected documents reveals a general trend of citation growth starting from 2014 with 27 citations (Figure 6). However, there were peak periods such as 2015 (58 citations), 2017 (72 citations), 2020 (70 citations), and 2021 (96 citations). Conversely, there were periods with relative decreases, like 2016 (56 citations), 2018 (62 citations), 2019 (50 citations), 2022 (77 citations), and up to June 2023 with 44 citations.

### 2.7. Analysis of Co-Occurrence and Keyword Density Analysis

**Figure 7:**
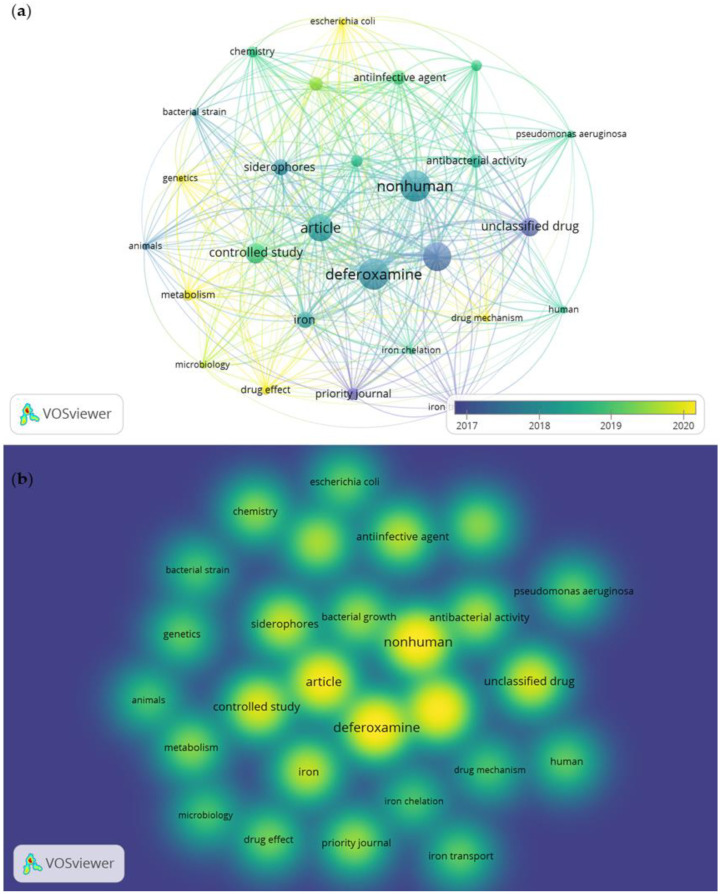
(**a**) Visualization of keyword co-occurrence. (**b**) Visualization of word density in publications on the antimicrobial activity of desferrioxamine, from 2012 to June of 2023.

The networks of keyword co-occurrence (a) and word density or “hotspots” (b) were elucidated through an analysis involving a combination of the most cited references, keyword co-occurrence, clusters, and bursts, primarily focusing on the antimicrobial activity of desferrioxamine. The system mapped 772 terms, with the minimum selection criterion being the occurrence of a term at least five times in the publications, resulting in 27 words, and among these, 7 keywords received special attention: “desferrioxamine”, “article”, “siderophore”, “controlled study”, “nonhuman”, “iron”, and “unclassified drug” (Figure 7).

In the visualization of the keyword co-occurrence analysis, the size of the colored circles indicates the frequency of occurrence of these words, and the lines connecting the nodes represent their co-occurrence in the same publication. The smaller the distance between two nodes, the higher the number of occurrences of the two keywords together. The hotspots are represented by the intensity of color and the size of the circle, where intense yellow stands out.

### 2.8. Analysis of Author Groups and Authors with Most Citations

**Figure 8:**
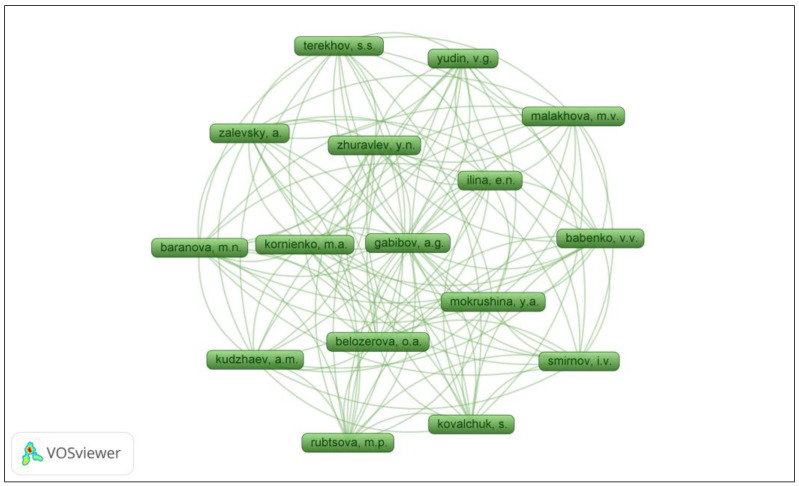
Visualization of the most cited authors in the field of the antimicrobial activity of desferrioxamine, from 2012 to June of 2023.

After analyzing the authors and co-authors, a total of 143 names were identified, considering a maximum of 25 authors per document and a minimum of 1 document per author. By examining the frequency of names, it was observed that the largest cluster of related authors consisted of 16 names (Figure 8).

## 3. Discussion

This bibliometric review study of the scientific literature published on the antimicrobial activity of desferrioxamine from 2012 to 2023 allowed us to visualize the most recent and globally relevant scientific productions related to the proposed topic. Through the analysis, we observed a scarcity of publications in indexed databases. Of the 41 articles initially selected (as shown in the flowchart), 17 were excluded for not meeting the inclusion criteria, leaving only 24 studies for bibliometric analysis within the proposed timeframe. Furthermore, there was an uneven distribution of publication years for these journals, with no publications in 2018 and almost three times as many in 2022 compared to the average of the other years. This uneven distribution may be strongly related to the onset of the SARS-CoV-2 pandemic, as it was particularly observed in 2021, leading to a significant decrease in the number of scientific publications on topics unrelated to COVID-19 [4].

Regarding the countries with the highest number of publications, the United States takes the lead. This can be explained by several factors, including greater incentives and investments in research compared to other countries, and primarily because the United States has historically provided ample space for scientific production, unlike many other countries, such as Brazil, which prioritizes professional education in key areas of knowledge rather than creating research opportunities [41].

Among the prominent articles, ten received a more significant number of citations, each publication ranging from 15 to 244 citations, for a total of 672 citations in the last 10 years for these ten articles analyzed. This high number of citations can be explained by the fact that most of these journals are in the field of medicine, indicating that these publications have gained significant international and multidisciplinary visibility on the topic [42,43].

Another striking result of this bibliometric review is the low impact of the publications as revealed by the h-index. However, when other citation metrics and impact factors were evaluated (CiteScore, SJR, and SNIP), focusing on desferrioxamine associated with siderophore and antimicrobial predictors, the most significant publications were found in journals dedicated to microbiology, immunology, and pharmacology. Several factors and barriers may explain this low impact of publications, such as the focus of journals on specific, innovative, and timely topics; low publication quality that prevents access to specialized and high-impact journals; lack of incentives for research in the field; the need for greater financial and technological investment to improve study quality, especially in developing countries; and the fact that a journal’s impact factor does not necessarily reflect the quality of an article [44]. In addition, research resources are often directed towards the development of high-cost technologies and treatments, making it difficult to implement study results in low-income countries [43,45].

In the word density analysis, the term “antimicrobial” was absent in both abstracts and titles. However, other terms such as “antibacterial activity” and “unclassified drug” may indicate similarity to the antimicrobial term. Siderophore desferrioxamine was the most prominent among the words. Siderophores are organic molecules that bind to iron with high affinity and specificity and perform various biological functions [46]. They have been studied in several areas, such as microbial ecology, where they can facilitate the cultivation of recalcitrant microorganisms; agriculture, where they can stimulate plant development; biological control, where they can act as antifungal or antibacterial agents; bioremediation, where they can mobilize or immobilize heavy metals; biosensing, where they can signal the presence of iron in various matrices; and medicine, where they can potentiate the action of antibiotics against multiresistant bacteria [47]. Research also reports the activities of siderophores with an antifungal effect, which is a mutual effect between iron and calcium in fungal pathogens, and the combination of calcium with an iron chelator could serve to improve antifungal therapy [48].

The presence of the words *Escherichia coli* and *Pseudomonas aeruginosa* near desferrioxamine was also verified, suggesting research with a potential siderophore effect for these two pathogens. No words related to the term mycobacteria were found. The use of appropriate terms that reflect the purpose of the research is paramount, as they effectively guide the reader and consolidate search strategies in databases [45].

In mapping the network of authors, we found that the largest network of researchers is composed of North American scholars, branching out into smaller groups while still maintaining key researchers across these groups. This quantitative consolidation forms a cohesive group of researchers. This phenomenon can be attributed to the regional proximity of researchers as well as the higher number of publications and citations originating from the same region/country [43].

This study presented limitations regarding the few references for data comparison. Thus, we found that there are few studies on the use of siderophores and the use of desferrioxamine as alternatives for host-directed therapies for mycobacteria. It is necessary to stimulate more research in the various areas of knowledge in the future, with funding and international cooperation, as only in this way will we obtain a solid base of information with more robust data regarding more effective treatments in the control of difficult-to-treat diseases, since these diseases have a negative impact on the quality of life of affected people.

## 4. Materials and Methods

### 4.1. Origin and Research Strategy

All data were collected in June 2023. The topic was desferrioxamine associated with siderophore and antimicrobial predictors; publications in peer-reviewed journals between 2012 and June of 2023 were retrieved from Scopus, WOS, CINAHL, EMBASE, WHO, and PubMed/Medline databases to conduct this research.

WOS (owned by Clarivate, formerly Thomson Reuters) and Scopus (owned by Elsevier) are the most well known sources of citation data with curation by subscribing institutions. They are also the most widely used databases for bibliometric analysis [15,16]. The combination of WOS and Scopus databases covers a wider range of scientific disciplines, a more comprehensive range of publication dates, and a wider range of countries while providing detailed citation analysis.

Cumulative Index to Nursing and Allied Health (CINAHL) involves rigorous curation of OA journals, resulting in a growing collection of 1253 global OA journals. Once validated and certified for inclusion, these OA journals undergo high-quality subject indexing and sophisticated, accurate full-text linking, covering titles published since 1937 (information collected from each database’s website—not included in the references).

Embase is a unique medical literature database that goes beyond content. By indexing full-text content from Emtree and specific search terms, it retrieves all relevant and current results, including information that may not be available in other databases.

The WHO Library is the world’s leading public health library. It provides access to WHO knowledge as well as to other sources of scientific literature produced worldwide. The resources and expertise of the WHO Library also bring scientific evidence and knowledge to low- and middle-income countries through a number of low-cost, high-impact initiatives.

PubMed is a free resource that supports the search and retrieval of biomedical and life sciences literature to improve global and individual health. The database contains over 35 million citations and abstracts from the biomedical literature. It does not include full-text journal articles; however, links to the full text are generally available if they are accessible from other sources, such as the publisher’s website or PubMed Central (PMC). PubMed has been publicly available online since 1996 and is developed and maintained by the National Center for Biotechnology Information (NCBI) at the National Library of Medicine (NLM), part of the National Institutes of Health (NIH).

**Figure 9:**
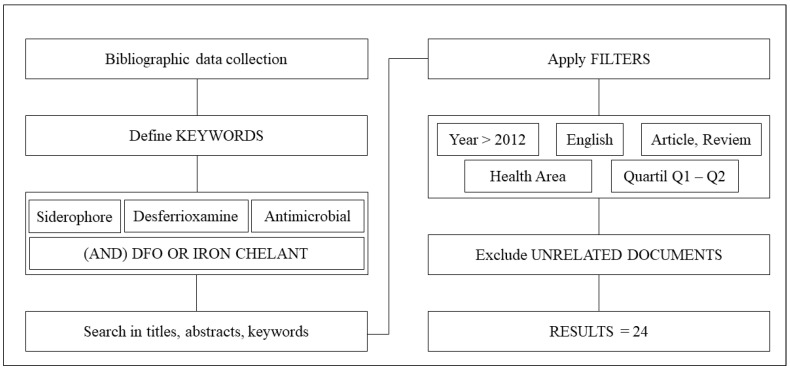
Bibliographic data collection process.

The search strategy used was as comprehensive as possible to identify all relevant publications. Keyword mapping was used to structure the search: “siderophores” AND “desferrioxamine” AND “antimicrobial”. Each article extracted from Scopus TM includes information on the authors, institutional affiliation, country, year of publication, journal of origin, title, abstract, keywords, and references. The literature search process is shown in Figure 9.

Literature quantity and publication trends were analyzed in terms of total publications, research types, research organizations, author contributions, journals, and funding. Publication quality was assessed by total citation frequency, average citations per article, h-index, CiteScore, SCImago Journal Rank (SjR), and Source Normalized Impact per Paper (SNIP). Related data such as the number of publications, citations, h-index, journal, references, and keywords were extracted and recorded as bibliometric indicators.

### 4.2. Preliminary Screening

Disagreements regarding inconsistent content were discussed and resolved. Two authors independently screened articles based on their titles to identify duplicate articles and articles that met the exclusion criteria (e.g., case studies, protocols, letters, books, and reports unrelated to the topic of interest). Articles that could not be judged to meet the inclusion criteria based on their titles alone were retained for the next screening stage [17], which was completed on 30 June 2023. Thus, inclusion and exclusion criteria were used to limit the age range of the articles reviewed (Table 4).

### 4.3. Access to Information

Citation information for all articles was exported from Scopus.

### 4.4. Bibliometric Analysis

This study conducted a quantitative descriptive analysis based on bibliometric analysis, and the data generated are presented in graphs and tables with results in absolute and percentage terms.

Bibliometric mapping and cluster analysis were performed using VOSviewer version 1.6.15 (Leiden University, the Netherlands), a software tool for constructing and visualizing bibliometric networks. Thematic maps were created using QGIS 3.32 from geocoding performed by measuring latitude and longitude on the website https://pt.batchgeo.com (accessed on 15 June 2023), using the DATUM SIRGAS 2000 geodetic reference system.

Bibliometric mapping visualizes the literary production in publications and citation information for a specific field. Cluster analysis uses different algorithms to detect the natural division of networks of research groups (clusters) based on similarity, allowing the visualization of co-authorship networks involving researchers, institutions, and countries [18].

### 4.5. Ethics

As this was an analysis of existing research, ethical approval was not required. No authors were contacted for further information about their publications.

## 5. Conclusions

The total number of publications on desferrioxamine and its biological activities has grown exponentially over the last decade. However, the proportion of articles on the topic remains low. Research groups worldwide are encouraged to focus their collaborative efforts on high-quality research and clinical trials.

## Figures and Tables

**Table 1:** Publications on the antimicrobial activity of desferrioxamine, from 2012 to June of 2023.

Year	Authors	Title	Journal
2023 [17]	Dvoretckaia, A., Egorova, T., Dzhuzha, A., (...), Demyanova, E., Korzhikova-Vlakh, E.	Polymyxin B Conjugates with Bio-Inspired Synthetic Polymers of Different Nature	*International Journal of Molecular Sciences*
2022 [18]	Shepherdson, E.M.F., Elliot, M.A.	Cryptic specialized metabolites drive *Streptomyces* exploration and provide a competitive advantage during growth with other microbes	*Proceedings of the National Academy of Sciences of the United States of America*
2022 [19]	Du, G.-F., Dong, Y., Fan, X., (...), Le, Y.-J., Yang, X.-Y.	Proteomic Investigation of the Antibacterial Mechanism of Cefiderocol against *Escherichia coli*	*Microbiology Spectrum*
2022 [20]	Pita-Grisanti, V., Chasser, K., Sobol, T., Cruz-Monserrate, Z.	Understanding the Potential and Risk of Bacterial Siderophores in Cancer	*Frontiers in Oncology*
2022 [21]	Naclerio, G.A., Onyedibe, K.I., Karanja, C.W., Aryal, U.K., Sintim, H.O.	Comparative Studies to Uncover Mechanisms of Action of *N*-(1,3,4-Oxadiazol-2-yl) benzamide Containing Antibacterial Agents	*ACS Infectious Diseases*
2022 [22]	Baranova, M.N., Kudzhaev, A.M., Mokrushina, Y.A., (...), Smirnov, I.V., Terekhov, S.S.	Deep Functional Profiling of Wild Animal Microbiomes Reveals Probiotic *Bacillus pumilus* Strains with a Common Biosynthetic Fingerprint	*International Journal of Molecular Sciences*
2022 [23]	Deflandre, B., Stulanovic, N., Planckaert, S., (...), Devreese, B., Rigali, S.	The virulome of *Streptomyces scabiei* in response to cello-oligosaccharide elicitors	*Microbial Genomics*
2021 [24]	Chan, D.C.K., Burrows, L.L.	Thiocillin and micrococcin exploit the ferrioxamine receptor of *Pseudomonas aeruginosa* for uptake	*Journal of Antimicrobial Chemotherapy*
2020 [25]	Dávila Costa, J.S., Hoskisson, P.A., Paterlini, P., Romero, C.M., Alvarez, A.	Whole genome sequence of the multi-resistant plant growth-promoting bacteria *Streptomyces* sp. Z38 with potential application in agroindustry and bio-nanotechnology	*Genomics*
2020 [26]	Phelan, J.J., McQuaid, K., Kenny, C., (...), O’Sullivan, M.J., Keane, J.	Desferrioxamine Supports Metabolic Function in Primary Human Macrophages Infected with *Mycobacterium tuberculosis*	*Frontiers in Immunology*
2020 [27]	Kidd, J.M., Abdelraouf, K., Nicolau, D.P.	Development of neutropenic murine models of iron overload and depletion to study the efficacy of siderophore-antibiotic conjugates	*Antimicrobial Agents and Chemotherapy*
2019 [28]	Saha, P., Xiao, X., Yeoh, B.S., (...), Kirimanjeswara, G.S., Vijay-Kumar, M.	The bacterial siderophore enterobactin confers survival advantage to *Salmonella* in macrophages	*Gut Microbes*
2017 [29]	Rana, N., Jesse, H.E., Tinajero-Trejo, M., (...), Schatzschneider, U., Poole, R.K.	A manganese photosensitive tricarbonyl molecule [Mn(CO)3(tpa-κ3N)]Br enhances antibiotic efficacy in a multi-drug-resistant *Escherichia coli*	*Microbiology* (United Kingdom)
2017 [30]	Huayhuaz, J.A.A., Vitorino, H.A., Campos, O.S., (...), Kaneko, T.M., Espósito, B.P.	Desferrioxamine and desferrioxamine-caffeine as carriers of aluminum and gallium to microbes via the Trojan Horse Effect	*Journal of Trace Elements in Medicine and Biology*
2017 [31]	Thyagarajan, S.L., Ramanathan, G., Singaravelu, S., (...), Perumal, P.T., Sivagnanam, U.T.	Microbial Siderophore as MMP inhibitor: An interactive approach on wound healing application	*Wound Medicine*
2016 [32]	Pupin, M., Esmaeel, Q., Flissi, A., (...), Jacques, P., Leclère, V.	Norine: A powerful resource for novel nonribosomal peptide discovery	*Synthetic and Systems Biotechnology*
2015 [33]	Kishimoto, S., Nishimura, S., Hatano, M., Igarashi, M., Kakeya, H.	Total Synthesis and Antimicrobial Activity of Chlorocatechelin A	*Journal of Organic Chemistry*
2015 [34]	Jayasinghe, S., Siriwardhana, A., Karunaratne, V.	Natural iron sequestering agents: Their roles in nature and therapeutic potential	*International Journal of Pharmacy and Pharmaceutical Sciences*
2014 [35]	Frangipani, E., Bonchi, C., Minandri, F., Imperi, F., Visca, P.	Pyochelin potentiates the inhibitory activity of gallium on *Pseudomonas aeruginosa*	*Antimicrobial Agents and Chemotherapy*
2014 [36]	Górska, A., Sloderbach, A., Marszałł, M.P.	Siderophore-drug complexes: Potential medicinal applications of the ‘Trojan horse’ strategy	*Trends in Pharmacological Sciences*
2014 [37]	Farkas, E., Szabó, O., Parajdi-Losonczi, P.L., Balla, G., Pócsi, I.	Mn(II)/Mn(III) and Fe(III) binding capability of two *Aspergillus fumigatus* siderophores, desferricrocin and N′, N″, N‴-triacetylfusarinine C	*Journal of Inorganic Biochemistry*
2013 [38]	Fukushima, T., Allred, B.E., Sia, A.K., (...), Andersen, U.N., Raymond, K.N.	Gram-positive siderophore-shuttle with iron-exchange from Fe-siderophore to apo-siderophore by *Bacillus cereus* YxeB	*Proceedings of the National Academy of Sciences of the United States of America*
2013 [39]	Traxler, M.F., Watrous, J.D., Alexandrov, T., Dorrestein, P.C., Kolter, R.	Interspecies interactions stimulate diversification of the *Streptomyces coelicolor* secreted metabolome	*mBio*
2012 [40]	Weiss, G., Fritsche, G., Nairz, M., Libby, S.J., Fang, F.C.	Slc11a1 (Nramp1) impairs growth of *Salmonella enterica* serovar *typhimurium* in macrophages via stimulation of lipocalin-2 expression	*Journal of Leukocyte Biology*

**Table 2:** Citations of the top 10 publications on the antimicrobial activity of desferrioxamine, from 2012 to June of 2023.

Article Title	Journal	Citations						
		<2019	2019	2020	2021	2022	2023	Total
		306	60	71	102	85	48	672
Interspecies interactions stimulate diversification of the Streptomyces coelicolor secreted metabolome	*MBio*	151	20	19	28	18	08	244
Siderophore-drug complexes: Potential medicinal applications of the ‘Trojan horse’ strategy	*Trends in Pharmacological Sciences*	54	12	13	18	13	07	117
Slc11a1 (Nramp1) impairs growth of salmonella enterica serovar typhimurium in macrophages via stimulation of lipocalin-2 expression	*Journal of Leukocyte Biology*	39	3	2	7	3	1	55
Gram-positive siderophore-shuttle with iron-exchange from Fe-siderophore to apo-siderophore by Bacillus cereus YxeB	*Proceedings of the National Academy of Sciences of the USA*	24	5	3	5	4	4	45
Pyochelin potentiates the inhibitory activity of gallium on Pseudomonas aeruginosa	*Antimicrobial Agents and Chemotherapy*	17	5	3	8	6	4	43
Norine: A powerful resource for novel nonribosomal peptide discovery	*Synthetic and Systems Biotechnology*	7	7	5	2	4	0	25
A manganese photosensitive tricarbonyl molecule [Mn(CO)3(tpa-κ3N)]Br enhances antibiotic efficacy in a multi-drug-resistant escherichia coli	*Microbiology*	3	2	8	3	6	2	24
Desferrioxamine Supports Metabolic Function in Primary Human Macrophages Infected with *Mycobacterium tuberculosis*	*Frontiers in Immunology*	-	-	3	9	6	3	21
The bacterial siderophore enterobactin confers survival advantage to Salmonella in macrophages	*Gut Microbes*	-	-	5	4	5	5	19
Desferrioxamine and desferrioxamine-caffeine as carriers of aluminum and gallium to microbes via the Trojan Horse Effect	*Journal of Trace Elements in Medicine and Biology*	2	3	3	5	1	1	15

**Table 3:** The top 10 institutions and the number of publications researching the antimicrobial activity of desferrioxamine, from 2012 to June of 2023.

Rank	Institution	Number of Papers	Citation	H-Index	Country
1	National Institute of Allergy and Infectious Diseases	4	345	3	USA
2	National Institutes of Health	2	19	1	USA
3	Natural Sciences and Engineering Research Council of Canada	2	9	2	Canada
4	Agencia Nacional de Promoción Científica y Tecnológica	1	7	1	Argentina
5	Beatson Institute for Cancer Research	1	21	1	United Kingdom
6	Biotechnology and Biological Sciences Research Council	1	24	1	United Kingdom
7	Consejo Nacional de Investigaciones Científicas y Técnicas	1	12	1	Argentina
8	Crohn’s and Colitis Foundation of America	1	19	1	USA
9	Duke University	1	0	0	USA
10	Fonds De La Recherche Scientifique—FNRS	1	4	1	Belgium

**Table 4:** Inclusion and exclusion criteria.

Inclusion Criteria	Exclusion Criteria
✓ Peer-reviewed journal articles ✓ Research articles ✓ Reviews (narrative and systematic) ✓ Clinical studies ✓ Theoretical articles ✓ Articles in English ✓ Abstract available ✓ Articles on DFO and *Mycobacteria* ✓ DFO as the topic of interest	✓ Dissertations ✓ Letters ✓ Conference abstracts ✓ Case studies ✓ Protocols ✓ Books ✓ Unrelated articles or those whose main outcome was not DFO

## Data Availability

Data sharing is not applicable.

## References

[1] Troesch A., Nguyen H., Miyada C.G., Desvarenne S., Gingeras T.R., Kaplan P.M., Cros P., Mabilat C. (1999). *Mycobacterium* species identification and rifampin resistance testing with high-density DNA probe arrays. J. Clin. Microbiol..

[2] Johansen M.D., Herrmann J.L., Kremer L. (2020). Non-tuberculous mycobacteria and the rise of *Mycobacterium abscessus*. Nat. Rev. Microbiol..

[3] Gordon S.V., Parish T. (2018). Microbe Profile: *Mycobacterium tuberculosis*: Humanity’s deadly microbial foe. Microbiology.

[4] Gao J., Yin Y., Myers K.R., Lakhani K.R., Wang D. (2021). Potentially long-lasting effects of the pandemic on scientists. Nat. Commun..

[5] Richard K.L., Kelley B.R., Johnson J.G. (2019). Heme uptake and utilization by gram-negative bacterial pathogens. Front. Cell. Infect. Microbiol..

[6] Pham D.D., Fattal E., Tsapis N. (2015). Pulmonary drug delivery systems for tuberculosis treatment. Int. J. Pharm..

[7] Johnston J.C., Cooper R., Menzies D. (2022). Chapter 5: Treatment of tuberculosis disease. Can. J. Respir. Crit. Care Sleep Med..

[8] Kapp E., Joubert J., Sampson S.L., Warner D.F., Seldon R., Jordaan A., de Vos M., Sharma R., Malan S.F. (2021). Antimycobacterial Activity, Synergism, and Mechanism of Action Evaluation of Novel Polycyclic Amines against. Mycobacterium tuberculosis. Adv. Pharmacol. Pharm. Sci..

[9] Ezzeddine Z., Ghssein G. (2023). Towards new antibiotics classes targeting bacterial metallophores. Microb. Pathog..

[10] Ghssein G., Ezzeddine Z. (2022). The Key Element Role of Metallophores in the Pathogenicity and Virulence of *Staphylococcus aureus*: A Review. Biology.

[11] Marchetti M., De Bei O., Bettati S., Campanini B., Kovachka S., Gianquinto E., Spyrakis F., Ronda L. (2020). Iron Metabolism at the Interface between Host and Pathogen: From Nutritional Immunity to Antibacterial Development. Int. J. Mol. Sci..

[12] Cassat J.E., Skaar E.P. (2013). Iron in infection and immunity. Cell Host Microbe.

[13] Bosne-David S., Bricard L., Ramiandrasoa F., DeRoussent A., Kunesch G., Andremont A. (1997). Evaluation of growth promotion and inhibition from mycobactins and nonmycobacterial siderophores (Desferrioxamine and FR160) in *Mycobacterium aurum*. Antimicrob. Agents Chemother..

[14] Cahill C., O’Connell F., Gogan K.M., Cox D.J., Basdeo S.A., O’Sullivan J., Gordon S.V., Keane J., Phelan J.J. (2021). The Iron Chelator Desferrioxamine Increases the Efficacy of Bedaquiline in Primary Human Macrophages Infected with BCG. Int. J. Mol. Sci..

[15] Lau C.K., Krewulak K.D., Vogel H.J. (2016). Bacterial ferrous iron transport: The Feo system. FEMS Microbiol. Rev..

[16] Zyoud S.H., Fuchs-Hanusch D. (2017). A bibliometric-based survey on AHP and TOPSIS techniques. Expert Syst. Appl..

[17] Dvoretckaia A., Egorova T., Dzhuzha A., Levit M., Sivtsov E., Demyanova E., Korzhikova-Vlakh E. (2023). Polymyxin B Conjugates with Bio-Inspired Synthetic Polymers of Different Nature. Int. J. Mol. Sci..

[18] Shepherdson E.M.F., Elliot M.A. (2022). Cryptic specialized metabolites drive Streptomyces exploration and provide a competitive advantage during growth with other microbes. Proc. Natl. Acad. Sci. USA.

[19] Du G.F., Dong Y., Fan X., Yin A., Le Y.J., Yang X.Y. (2022). Proteomic Investigation of the Antibacterial Mechanism of Cefiderocol against Escherichia coli. Microbiol. Spectr..

[20] Pita-Grisanti V., Chasser K., Sobol T., Cruz-Monserrate Z. (2022). Understanding the Potential and Risk of Bacterial Siderophores in Cancer. Front. Oncol..

[21] Naclerio G.A., Onyedibe K.I., Karanja C.W., Aryal U.K., Sintim H.O. (2022). Comparative Studies to Uncover Mechanisms of Action of N-(1,3,4-Oxadiazol-2-yl) benzamide Containing Antibacterial Agents. ACS Infect. Dis..

[22] Baranova M.N., Kudzhaev A.M., Mokrushina Y.A., Babenko V.V., Kornienko M.A., Malakhova M.V., Yudin V.G., Rubtsova M.P., Zalevsky A., Belozerova O.A. (2022). Deep Functional Profiling of Wild Animal Microbiomes Reveals Probiotic Bacillus pumilus Strains with a Common Biosynthetic Fingerprint. Int. J. Mol. Sci..

[23] Deflandre B., Stulanovic N., Planckaert S., Anderssen S., Bonometti B., Karim L., Coppieters W., Devreese B., Rigali S. (2022). The virulome of Streptomyces scabiei in response to cello-oligosaccharide elicitors. Microb. Genom..

[24] Chan D.C.K., Burrows L.L. (2021). Thiocillin and micrococcin exploit the ferrioxamine receptor of *Pseudomonas aeruginosa* for uptake. J. Antimicrob. Chemother..

[25] Dávila Costa J.S., Hoskisson P.A., Paterlini P., Romero C.M., Alvarez A. (2020). Whole genome sequence of the multi-resistant plant growth-promoting bacteria *Streptomyces* sp. Z38 with potential application in agroindustry and bionanotechnology. Genomics.

[26] Phelan J.J., McQuaid K., Kenny C., Gogan K.M., Cox D.J., Basdeo S.A., O’Leary S., Tazoll S.C., Maoldomhnaigh C.Ó., O’Sullivan M.P. (2020). Desferrioxamine Supports Metabolic Function in Primary Human Macrophages Infected with *Mycobacterium tuberculosis*. Front. Immunol..

[27] Kidd J.M., Abdelraouf K., Nicolau D.P. (2020). Development of neutropenic murine models of iron overload and depletion to study the efficacy of siderophoreantibiotic conjugates. Antimicrob. Agents Chemother..

[28] Saha P., Xiao X., Yeoh B.S., Chen Q., Katkere B., Kirimanjeswara G.S., VijayKumar M. (2019). The bacterial siderophore enterobactin confers survival advantage to *Salmonella* in macrophages. Gut Microbes.

[29] Rana N., Jesse H.E., Tinajero-Trejo M., Butler J.A., Tarlit J.D., Von Und Zur Muhlen M.L., Nagel C., Schatzschneider U., Poole R.K. (2017). A manganese photosensitive tricarbonyl molecule [Mn(CO)3(tpa-κ3N)]Br enhances antibiotic efficacy in a multi-drug-resistant *Escherichia coli*. Microbiology.

[30] Huayhuaz J.A.A., HVitorino A., Campos O.S., Serrano S.H.P., Kaneko T.M., Espósito B.P. (2017). Desferrioxamine and desferrioxamine-caffeine as carriers of aluminum and gallium to microbes via the Trojan Horse Effect. J. Trace Elem. Med. Biol..

[31] Thyagarajan S.L., Ramanathan G., Singaravelu S., Kandhasamy S., Perumal P.T., Sivagnanam U.T. (2017). Microbial Siderophore as MMP inhibitor: An interactive approach on wound healing application. Wound Med..

[32] Pupin M., Esmaeel Q., Flissi A., Dufresne Y., Jacques P., Leclère V. (2016). Norine: A powerful resource for novel nonribosomal peptide discovery. Synth. Syst. Biotechnol..

[33] Kishimoto S., Nishimura S., Hatano M., Igarashi M., Kakeya H. (2015). Total Synthesis and Antimicrobial Activity of Chlorocatechelin A. J. Org. Chem..

[34] Jayasinghe S., Siriwardhana A., Karunaratne V. (2015). Natural iron sequestering agents: Their roles in nature and therapeutic potential. Int. J. Pharm. Pharm. Sci..

[35] Frangipani E., Bonchi C., Minandri F., Imperi F., Visca P. (2014). Pyochelin potentiates the inhibitory activity of gallium on Pseudomonas aeruginosa. Antimicrob. Agents Chemother..

[36] Górska A., Sloderbach A., Marszall M.P. (2014). Siderophore-drug complexes: Potential medicinal applications of the ‘Trojan horse’ strategy. Trends Pharmacol. Sci..

[37] Farkas E., Szabó O., Parajdi-Losonczi P.L., Balla G., Pócsi I. (2014). Mn(II)/Mn(III) and Fe(III) binding capability of two *Aspergillus fumigatus* siderophores, desferricrocin and N′, N″, N‴- triacetylfusarinine C. J. Inorg. Biochem..

[38] Fukushima T., Allred B.E., Sia A.K., Nichiporuk R., Andersen U.N., Raymond K.N. (2013). Gram-positive siderophore-shuttle with iron-exchange from Fesiderophore to apo-siderophore by Bacillus cereus YxeB. Proc. Natl. Acad. Sci. USA.

[39] Traxler M.F., Watrous J.D., Alexandrov T., Dorrestein P.C., Kolter R. (2013). Interspecies interactions stimulate diversification of the *Streptomyces coelicolor* secreted metabolome. mBio.

[40] Weiss G., Fritsche G., Nairz M., Libby S.J., Fang F.C. (2012). Slc11a1 (Nramp1) impairs growth of *Salmonella enterica* serovar *typhimurium* in macrophages via stimulation of lipocalin-2 expression. J. Leukoc. Biol..

[41] Cadillo C.D.M. (2014). The Role of Patents in Latin American Development: Models of Protection of Pharmaceutical Patents and Access to Medicines in Brazil, Chile and Venezuela.

[42] Garcia J.B.S., de Moraes É.B., Neto J.O.B. (2021). A Bibliometric Analysis of Published Literature in Postoperative Pain in Elderly Patients in Low- and Middle-Income Countries. J. Clin. Med..

[43] Bonorino C. (2015). Scientific Research in Brazil is Undervalued. GZH Porto Alegre. https://gauchazh.clicrbs.com.br/porto-alegre/noticia/2015/08/pesquisa-cientifica-no-brasil-e-menosprezada-4825155.html.

[44] Zhao Y., Zhang Z., Guo S., Feng B., Zhao X., Wang X., Wang Y. (2021). Bibliometric Analysis of Research Articles on Pain in the Elderly Published from 2000 to 2019. J. Pain Res..

[45] Doğan G., Karaca O. (2020). A bibliometric analysis of the field of anesthesia during 2009–2018. Braz. J. Anesthesiol..

[46] Kramer J., Özkaya Ö., Kümmerli R. (2020). Bacterial siderophores in community and host interactions. Nat. Rev. Microbiol..

[47] Saha M., Sarkar S., Sarkar B., Sharma B.K., Bhattacharjee S., Tribedi P. (2016). Microbial siderophores and their potential applications: A review. Environ. Sci. Pollut. Res. Int..

[48] Ye J., Wang Y., Li X., Wan Q., Zhang Y., Lu L. (2022). Synergistic Antifungal Effect of a Combination of Iron Deficiency and Calcium Supplementation. Microbiol. Spectr..

